# Association between *TLR4* and *TLR9* Gene Polymorphisms with Development of Pulmonary Tuberculosis in Zahedan, Southeastern Iran

**DOI:** 10.1155/2013/534053

**Published:** 2013-05-26

**Authors:** Danial Jahantigh, Saeedeh Salimi, Roya Alavi-Naini, Abolfazl Emamdadi, Hamid Owaysee Osquee, Farzaneh Farajian Mashhadi

**Affiliations:** ^1^Cellular and Molecular Research Center, Zahedan University of Medical Sciences, Zahedan, Iran; ^2^Department of Biochemistry, School of Medicine, Zahedan University of Medical Sciences, Zahedan, Iran; ^3^Infectious Diseases and Tropical Medicine Research Center, Zahedan University of Medical Sciences, Zahedan, Iran; ^4^Infectious Diseases Department, Zabol University of Medical Sciences, Zabol, Iran; ^5^Department of Pharmacology, School of Medicine, Zahedan University of Medical Sciences, Zahedan, Iran

## Abstract

Some evidence suggests that a variety of genetic factors contribute to development of the tuberculosis (TB). *TLR4* and *TLR9* have been proposed as susceptibility genes for TB. This study was performed in 124 newly diagnosed TB cases and 149 healthy controls in a TB-endemic region of Iran. The *TLR4* genes Asp299Gly, Thr399Ile, and *TLR9* gene T-1486C polymorphisms were amplified by polymerase chain reaction (PCR) and then detected by PCR-restriction fragment length polymorphism (RFLP). The frequencies of the mutant alleles of *TLR4* Arg299Gly, Thr399Ile, and *TLR9* T-1486C polymorphisms were 0.8 *versus* 0.1, 5.6 *versus* 3, and 28.6 *versus* 25.2 in patients and controls, respectively, that were not significant. The synergic effect of TI,II/CC genotypes for *TLR4* Thr399Ile and *TLR9* T-1486C polymorphisms showed increased risk of PTB susceptibility. In conclusion, no significant relation was found between *TLR4* and *TLR9* polymorphisms alone and PTB. However, synergic effects of *TLR4* Thr399Ile and *TLR9*-1486T/C polymorphisms might increase risk of PTB.

## 1. Introduction

Tuberculosis (TB), an ancient and devastating disease, is still a significant public health problem. It is an infectious bacterial disease caused by *Mycobacterium tuberculosis* (MT) which affects more than 9 million people each year and causes about 1.7 million deaths per year [1]. 

The incidence of TB in Iran was reported to be 13.7 per 100 000 population in 2011 [2]. However, in Sistan-Baluchestan Province in southeastern Iran, the incidence was much higher due to neighboring with high TB prevalence countries such as Afghanistan and Pakistan. In 2011, TB incidence was 43/100 000 in Zahedan, the center of Sistan-Baluchistan [2]. 

Most of the population in this region comprises Persian, Baluch, and Afghan ethnic groups.

Despite infection of more than one-third of the world's population with *M. tuberculosis*, only 5% to 10% of infected population has a risk of developing active tuberculosis and clinical symptoms and signs of the disease, suggesting that host defense factors influence development of active disease. Although certain data such as familial clustering, increased concordance rates among monozygotic twins, and familial differences in incidence strongly support genetic predisposition as a key element in susceptibility, the biological mechanisms underlying genetic variations remain mostly unknown. Several studies have been investigated on associations between tuberculosis and gene polymorphisms, and some of these candidate genes have shown significant association with TB susceptibility [1–3]. 

Toll-like receptors (TLRs) are a class of pattern recognition molecules that play a unique function in the innate immune system [4]. This system is the first line of defense against microorganisms that initiate cellular signal in response to pathogen-associated molecular patterns (PAMPs) and induce expression of genes involved in the inflammatory process, therefore it plays a crucial role initiating and directing the adaptive immune system [5, 6]. 

Ten functional TLR members (TLR1–TLR13) have been identified in humans. TLR4 is activated by bacterial lipopolysaccharides (LPSs) and has critical role in initiation of innate immune system [4–6]. TLR4 has been shown to activate IL-1 receptor-associated kinase in response to a variety of PAMPs, such as Gram-negative enterobacterial LPS [7]. 

The *TLR4* gene is located on chromosome 9q32-33, spans approximately 13 kb, and contains three exons that encode a 222-amino acid protein. Several studies show that synergic effect of two variants of *TLR4* gene, Asp299Gly (rs4986790) and Thr399Ile (rs4986791) which both are encoded within the fourth exon of the *TLR4* gene, are associated with an endotoxin-hyporesponsive phenotype. Moreover, the Asp299Gly polymorphism is associated with airway hyporesponsiveness in either human primary airway epithelial cells or alveolar macrophages obtained from individuals with these *TLR4* mutations [8–11]. 

TLR9, an endosomal localized receptor on B cells, plasmacytoid dendritic cells (pDCs), and monocytes/macrophages, recognizes unmethylated nucleic acid motifs, especially Cytosine-phosphate-Guanine (CpG) motifs, in bacterial DNA [12], and it is one of the most important receptors in the initiation of protective immunity against intracellular pathogens by activation signaling cascade of intracellular receptor signaling [13, 14]. *TLR9* encoding gene is located on chromosome 3p21.3. It spans approximately 5 kb and contains two exons, the second of which is the major coding region [12–14]. Twenty SNPs have been identified for *TLR9*, which -1486T/C (rs187084) in the promoter region in intron 1 is one of the most important SNPs [15, 16]. 

*TLR4* and *TLR9* gene polymorphisms have been extensively studied for their association with susceptibility or resistance to many infections and diseases [17–22]. Therefore, the association between progress and severity of infectious diseases with *TLR4* [23–27] and *TLR9* [28, 29] gene polymorphisms has been confirmed. 

It has been shown that *TLR4* and *TLR9* have critical roles in the recognition of MT, and they are necessary for development of an adequate immune response against MT [30–33]. *TLR*-knockout mouse studies indicate that *TLR4* and *TLR9 *contribute to host resistance to *M. tuberculosis *infection [34]. Moreover, associations between genetic variations of *TLR4* and *TLR9* and TB have been reported [35–42]. There have been no reports about the association of *TLR4* and *TLR9* with TB in Iran so far. The aim of the present study was to investigate the potential association between pulmonary TB, a TB infection of the lungs, and three SNPs in *TLR4* and *TLR9* genes in southeastern Iranian population, Zahedan.

## 2. Materials and Methods

### 2.1. Sample Collection

The project was approved by Zahedan University of Medical Sciences Ethics Committee. This case-control study was conducted prospectively at Boo-Ali Hospital, Zahedan, southeastern Iran, from March 2010 to May 2011. 

The diagnosis of different clinical forms of active pulmonary and extrapulmonary TB was established according to the criteria defined by the American Thoracic Society for diagnosis of disease caused by MT [43]. Patients included were clinically and radiologically diagnosed for pulmonary tuberculosis and confirmed by sputum smear and culture for *M. tuberculosis*. TB patients with other comorbidities such as myocardial infarction, liver cirrhosis, acute pancreatitis, and septic shock were excluded. The inclusion criteria for control group were absence of clinical symptoms of active PTB and normal CXR. Also the controls had no medical history of TB, other infectious and autoimmune diseases, cancer and other diseases affecting host immunity.

Peripheral blood samples were collected in EDTA tubes from 124 pulmonary tuberculosis (PTB) patients and 149 unrelated healthy controls, with no history of TB or other immune diseases. All the participants signed the written informed consent. 

### 2.2. Genotyping

Genomic DNA was extracted from peripheral blood lymphocytes using the commercial available kit (Roche, Germany) in accordance with the manufacturer's instructions, and extracted DNA was stored at −20°C until analyzing. The alleles of *TLR4* and *TLR9* gene polymorphisms were detected using polymerase chain reaction-restriction fragment length polymorphism (PCR-RFLP) according to previously described methods [29, 44]. 

PCR reactions were performed in a 25 **μ**L reaction volume containing 200 ng genomic DNA, 25 *ρ*M of each primer, 2.5 mM deoxyribonucleoside triphosphates (dNTPs), 1.5 mM MgCl_2_, and 1 U thermostable Taq DNA polymerase (Fermentas, Lithuania). Reactions were run on MyCycler Thermal cycler, BIO-RAD PCR system (BIO-RAD Co., USA), using the following conditions: initial denaturation at 95°C for 5 minutes followed by 35 cycles of denaturation at 94°C for 30 seconds, annealing at 61°C for 30 seconds, and extention at 72°C for 1 minute. A final extension step was at 72°C for 10 minutes. The PCR products were held at 4°C until analysis. The primers and PCR conditions used to detect TLR variants were listed in Table 1. PCR products were electrophoresed on agarose gel and visualized by ethidium bromide staining.

The PCR products of *TLR4* Arg299Gly, *TLR4* Thr399Ile, and *TLR9* T-1486C polymorphisms were digested overnight at 37°C with 10 U of NcoI, HinfI, and BslI restriction endonucleases (Fermentas, Lithuania), respectively. 

The wild-type allele of *TLR4* Arg299Gly polymorphism (A allele) was 249 bp size and had no NcoI cleavage site, whereas mutant allele (G allele) digested to 223 and 26 bp fragments. The fragment sizes for carriers of the *TLR4* Thr399Ile polymorphic allele were decreased from 406 bp (C allele) to 377 and 29 bp fragments (T allele). The 558 bp PCR product of *TLR9* T-1486C polymorphism digested to 264, 145, 126, and 23 bp fragments for T allele and to 264, 115, 126, 30, and 23 bp fragments for C allele. Digested samples were separated by electrophoresis on 2% agarose gel and visualized by ethidium bromide staining.

### 2.3. Statistical Analysis

All data were analyzed using SPSS software version 15. The differences in clinical characteristics between groups were examined by *χ* ^2^ test or an independent student *t*-test whenever appropriate. Allele frequencies were estimated by the gene counting method. Differences in frequency of alleles and genotypes were analyzed using the *χ* ^2^ test or Fisher's exact test. The odds ratio (OR) and 95% confidence intervals (CIs) were estimated. The *χ* ^2^ test was used to analyze the deviation of genotype distribution from the Hardy-Weinberg equilibrium. *P* < 0.05 was considered statistically significant.

## 3. Results

Table 2 shows the demographic and clinical characteristics of the 124 TB patients and 149 healthy controls. Because this was a matched case-control study, the cases and controls did not differ significantly by sex, age, and ethnic characteristics. 

The frequency of smokers was higher in TB patients (31 percent) compared to controls (17.5 percent; *P* = 0.008). 

### 3.1. Asp299Gly and The399Ile TLR4 Genotyping

There were no significant deviations from Hardy-Weinberg equilibrium for genotype frequencies of these SNPs in cases or controls. As shown in Table 3, distribution of alleles and genotypes of Asp299Gly polymorphism was not significantly different between two groups. The frequency of 299Gly allele was 0.8 percent in the patients compared with 1 percent in control group (*χ* ^2^ = 0.06; *P* = 0.6; OR (95% CI) = 1.3 (0.2–7.6)). The frequency of heterozygote genotype (AG) was not significantly different between patients and controls (1.6% versus 2; *P* = 0.6). Also, homozygous state of mutant allele (GG genotype) was not observed in both pulmonary TB patients and controls. 

The distribution of Thr399Ile polymorphism alleles and genotypes in PTB patients and healthy controls is also shown in Table 3. The frequency of the mutant T (399 Ile) allele was 5.6 percent in the patients as compared with 3 percent in controls and were not significantly different (*χ* ^2^ = 2.3; *P* = 0.1; OR (95% CI) = 1.9 (0.8–4.5)). No significant differences were found between PTB patients and healthy controls for the CT (8.1 versus 4.7 percent, *P* = 0.18) and TT genotypes (1.6 versus 0.7 percent, *P* = 0.4).

### 3.2. T-1486C TLR9 Genotyping

The frequencies of *T-1486C TLR9* genotypes and alleles were not significantly different between pulmonary TB patients and the controls (Table 4). The mutant allele frequency of *TLR9 T-1486C *was 25.2 percent in the control compared with 28.6 percent in patients group (*χ* ^2^ = 0.8; *P* = 0.2; OR (95% CI) = 1.2 (0.8–1.8)). The proportion of heterozygous TC genotype in control group was 39.6 percent while in pulmonary TB patients was 41 percent (*χ* ^2^ = 0.2; *P* = 0.4). Moreover, the frequencies of CC genotype were 5.4 and 8 percent in controls and PTB patients, respectively. 

### 3.3. Combination Effect of TLR4 Thr399Ile and TLR9 T-1486C Polymorphisms on PTB Susceptibility

Regarding the low frequency of *TLR4* Asp299Gly polymorphism in the study population, we analyzed only the combination effect of *TLR4* Thr399Ile and *TLR9* T-1486C polymorphisms on TB susceptibility.  Table 5 shows the combined genotype distribution for both polymorphisms. The heterozygous, homozygous/heterozygous, homozygous state for *TLR4* Thr399Ile and *TLR9* T-1486C polymorphisms showed a 1.7-fold increased risk of pulmonary TB, with a 95 percent confidence limit of 0.6–4.6 and a *P* value of 0.23 which was not significant. Moreover, the heterozygous, homozygous/homozygous state for both polymorphisms, respectively, showed 7.5-fold increased risk of pulmonary TB, with a 95 percent confidence limit of 0.84–66.7 and a *P* value of 0.047 which was slightly significant.

## 4. Discussion

In this study, there was not any evidence for a significant association of *TLR4* Asp299Gly and Thr399Ile and *TLR9 T-1486C* polymorphisms with pulmonary TB risk, but the combination effect of TI,II/CC genotypes for *TLR4* Thr399Ile and *TLR9* T-1486C polymorphisms showed 7.5-fold increased risk of pulmonary TB which was slightly significant (*P* = 0.047). Pulmonary tuberculosis is endemic in southeastern Iranian territories, particularly in the northern regions of Sistan and Baluchistan Province, Zahedan. *TLR4* and *TLR9* have shown biological relevance and linkage disequilibrium with the other polymorphisms [8–11, 14]. Therefore, to determine whether *TLR4* and *TLR9* are associated with susceptibility to pulmonary TB in the current study, two common *TLR4* polymorphisms, Asp299Gly and Thr399Ile, and one well-known* TLR9 *gene polymorphism,* T-1486C, *were examined. 

The TLR4 is known to activate the NF-*κ*B via signal transduction involving various intracellular signaling systems and expression of various inflammatory cytokines [6, 7]. TLR-4 polymorphisms that prevent ligands binding would result in lower NF-*κ*B activation and subsequent NF-*κ*B dependent proinflammatory genes expression. Moreover, it is established that two variants of *TLR4 *gene, Asp299Gly and Thr399Ile, affected a ligand-binding region and a coreceptor-binding region, respectively. Therefore, these two polymorphisms appear to affect the extracellular domain of the TLR4 receptor and have been linked with inability to trigger LPS signaling in some cell types, including primary airway epithelial cells [8, 9]. Furthermore, *TLR4* Asp299Gly polymorphism was found to be associated with systemic inflammatory hyporesponsiveness after LPS inhalation [9, 10]. *TLR4* SNPs have shown an association with increased risk of many infections and diseases including mortality from systemic inflammatory response syndrome, severe acute infections, Gram-negative septic shock [10, 11], respiratory syncytial virus bronchiolitis [24], and ischemic stroke [25], as well as with the host susceptibility to autoimmune process in human [26]. 

Genetic variations of TLR9, as a key gene of innate immunity, could affect immunological downstream responses that are critically important for host defense or inflammatory disease pathogenesis. The roles played by *TLR9 *SNPs are unclear; however, Lazarus et al. in 2003 and Coban et al. in 2005 demonstrated that *TLR9 *T-1486C promoter SNP modifies the expression and consequently its function [15, 16]. 

Moreover, several investigators have studied the role of *TLR9 *genetic polymorphisms in major infection diseases, systemic lupus [22], type 2 diabetes and coronary artery disease [28], and malaria [29]. 

Several studies have demonstrated critical role of *TLR4* and *TLR9* in *M. tuberculosis* recognition and verified necessity of these TLRs for development of a protective response against MTB infection [30–34]. Variants in *TLR4*, Asp299Gly and Thr399Ile, and the *TLR9*-1486C/T were investigated for their association with susceptibility or resistance to pulmonary tuberculosis [35–42].

The prevalence of mutant alleles of TLR4 Asp299Gly and Thr399Ile polymorphisms in this study was similar to that reported previously in other Asian populations (almost 0%) [19–21] but significantly lower than in European Caucasians (3%–9%) [21, 22], North and South American (almost 4.5%) [23, 37], and African populations (7.5%–21%) [27, 29, 35]. 

Contrary to the present study, Najmi et al. in India [39] reported an association between *TLR4* Asp299Gly and Thr399Ile polymorphisms and susceptibility to pulmonary tuberculosis especially in severe form of the disease (*P* = 0.001). The differences between result of the current study and that by Najmi et al. [39] described the differences in the number of enrolled subjects and higher frequencies of both mutant alleles in Indian population. The results of our study are consistent with the reports of Zafra et al. in Colombia [23], Newport et al. in Gambia [35], Rosas-Taraco et al. in Mexico [38], and Velez et al. in USA [40]. 

In addition, Ferwerda et al. and Pulido et al. revealed a relation between TLR4 Asp299Gly polymorphism and active TB only in HIV-infected patients in Tanzania and Spain, respectively [36, 37].

In this study, the mutant allele frequency of *TLR9*-1486C/T polymorphism in controls was 25.2 percent, which is similar to that found previously in Chinese [28], Iranian [29], South Indian [41], and Indonesian females [42] populations, but not with the Japanese [22]. Similar to the present study, results of Selvaraj et al. [41] and Kobayashi et al. [42] did not support the association between *TLR9*-1486T/C polymorphism and the prevalence of pulmonary TB. 

The present study revealed the synergic effect of TI,II/CC genotypes of *TLR4* Thr399Ile and *TLR9* T-1486C polymorphisms with the increasing risk of pulmonary TB. This result was the first published report about synergic effect of these genes on pulmonary tuberculosis susceptibility. 

In conclusion, we demonstrated that *TLR4* Asp299Gly and Thr399Ile and *TLR9*-1486T/C polymorphisms were not associated with susceptibility to pulmonary tuberculosis. Moreover, the frequencies of *TLR4* 299Gly and 399Ile alleles were nearly rare in southeastern Iran both in the patients and in the healthy controls. Due to the relatively small number of patients and the possibility of racial differences, further investigations using a larger sample size and different ethnicities to confirm the present findings are necessary. 

## Figures and Tables

**Table 1:** Primers, PCR conditions, and restriction enzymes used for genotyping TLRs genes.

SNPs product	Primer sequence 5′-3′	AT	PCR product	RE
*TLR4 *				
*Arg299Gly *	F: GATTAGCATACTTAGACTACTACCTCCATG	60°	249 bp	NcoI
	R: GATCAACTTCTGAAAAAGCATTCCCAC			
*Thr399Ile *	F: GGTTGCTGTTCTCAAAGTGATTTTGGGAGAA	61°	406 bp	HinfI
	R: CCTGAAGACTGGAGAGTGAGTTAAATGCT			

*TLR9 *				
*T-1486C *	F: TTCATTCAGCCTTCACTCAG	55°	558 bp	BslI
	R: TCAAAGCCACAGTCCACAG			

F: forward primer; R: reverse primer; AT: annealing temperature; RE: restriction enzyme.

**Table 2:** Demographic characteristics of TB patients and controls.

	TB	Controls	*χ*²	*P* value	OR
	*n* = 124	*n* = 149			(95% CI)
Age (years)	51.1 ± 20	48.4 ± 14.7		0.2	
Sex (male/female)	48/76	50/99	0.8	0.2	
Smoking *n* (%)	38 (31)	26 (17.5)	6.6	0.008	2.1 (1.2–3.7)
Race *n* (%)			4.9	0.09	
Persian	48 (38.7)	39 (26.2)			
Balouch	72 (58.1)	104 (69.8)			
Afghan	4 (3.2)	6 (4)			

**Table 3:** *TLR4* SNPs allele and genotype frequencies in patients and controls.

*TLR4* polymorphism	PTB patients (*n* = %)	Controls (*n* = %)	*χ* ^ 2^	*P* value*	OR (95% CI)
Asp299Gly (A896G)					
AA	122 (98.4)	146 (98)			
AG	2 (1.6)	3 (2)	0.06	0.6	0.8 (0.13–4.9)
GG	0	0			

Total	124	149			

A (WT)	246 (99.2)	295 (99)			
G (Mut)	2 (0.8)	3 (1)	0.06	0.6	1.3 (0.2–7.6)

Thr399Ile (C1196T)					
CC	112 (90.3)	141 (94.6)			
TC	10 (8.1)	7 (4.7)	1.4	0.18	1.8 (0.7–4.9)
TT	2 (1.6)	1 (0.7)	0.6	0.4	2.5 (0.2–2.8)

Total	124	149			

C (WT)	234 (94.4)	289 (97)			
T (Mut)	14 (5.6)	9 (3)	2.3	0.1	1.9 (0.8–4.5)

*Compared with healthy controls. A corrected *P* value < 0.05 was considered significant.

**Table 4:** *TLR9*-1486T/C genotype and allele frequencies in patients and controls.

SNP	PTB patients (*n* = %)	Controls (*n* = %)	*χ* ^2^	*P* value*	OR (95% CI)
*TLR9* (T-1486C)					
TT	63 (51)	82 (55)			
TC	51 (41)	59 (39.6)	0.2	0.4	1.1 (0.7–1.9)
CC	10 (8)	8 (5.4)	1	0.2	1.6 (0.6–4.4)

Total	124	149			

T	177 (71.4)	223 (74.8)			
C	71 (28.6)	75 (25.2)	0.8	0.2	1.2 (0.8–1.8)

*Compared with healthy controls. A corrected *P* value < 0.05 was considered significant.

**Table 5:** Combined effect of both *TLR4* Thr399Ile and *TLR9* T-1486C polymorphisms in cases and controls.

*TLR4 *	*TLR9 *	Patients	Controls	*P* value	OR (95% CI)
Thr399Ile	T-1486C	*n* = 124	*n* = 149		
TT	TT	55	82	0.23	Ref = 1
TI, II	TC, CC	9	8	0.6	1.7 (0.6–4.6)
TI, II	CC	5	1	0.047	7.5 (0.84–66.7)
